# Modified nucleic acids: replication, evolution, and next-generation therapeutics

**DOI:** 10.1186/s12915-020-00803-6

**Published:** 2020-09-02

**Authors:** Karen Duffy, Sebastian Arangundy-Franklin, Philipp Holliger

**Affiliations:** MRC Laboratory of Molecular Biology, Cambridge Biomedical Campus, Francis Crick Avenue, Cambridge, CB2 0QH UK

## Abstract

Modified nucleic acids, also called xeno nucleic acids (XNAs), offer a variety of advantages for biotechnological applications and address some of the limitations of first-generation nucleic acid therapeutics. Indeed, several therapeutics based on modified nucleic acids have recently been approved and many more are under clinical evaluation. XNAs can provide increased biostability and furthermore are now increasingly amenable to in vitro evolution, accelerating lead discovery. Here, we review the most recent discoveries in this dynamic field with a focus on progress in the enzymatic replication and functional exploration of XNAs.

## Nucleic acids: natural and expanded functions

A heritable genetic system is a defining requirement for life. The natural nucleic acids, DNA and RNA, are exquisitely suited to store and propagate genetic information with sufficient stability and fidelity to support large genomes but also the flexibility to enable evolution. Nucleic acids are unique among biopolymers in that both accessible information and functional capacity coexist in a single molecule. Thus, function can be evolved at the molecular level yielding nucleic acid-based ligands and enzymes. In addition, nucleic acids fold through predictable and (to a large extent) programmable interactions, are highly water-soluble, and can be easily denatured and refolded. These advantages underpin their manifold uses in biotechnology, diagnostics, therapeutics, nanotechnology, material science, synthetic biology, and data storage.

The last four decades have seen the rise of nucleic acids as therapeutics [1], primarily in the form of antisense oligonucleotides (ASOs) [2], small interfering RNAs (siRNAs) [3], aptamers [4–6], microRNAs [7], mRNAs [8], and gene-editing guides [9]. Nucleic acids afford completely new modalities for therapeutic intervention (e.g., direct protein expression or specific and programmable modulation of gene expression) that cannot be easily accessed by other biologics or small molecule drugs. However, several challenges have thus far prevented nucleic acids from reaching their full potential as medicines including poor chemical and biological stability and narrow chemical diversity.

**Fig. 1:**
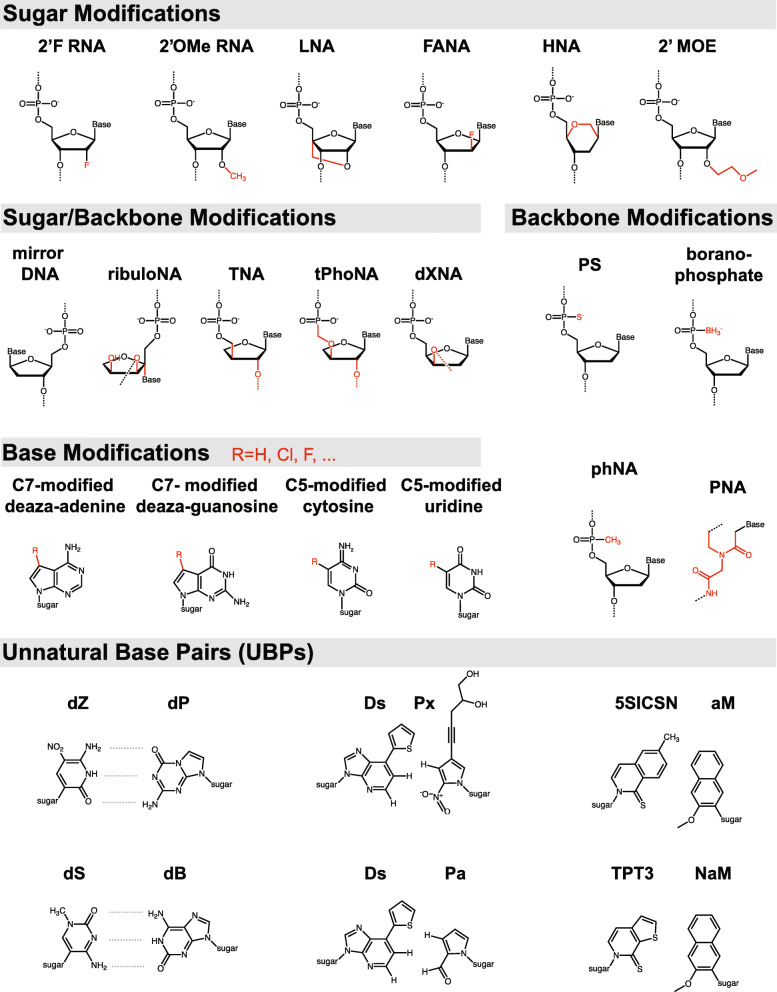
The chemical diversity of XNAs. XNAs are often categorized by the component of the nucleotide (sugar, backbone, or base) carrying a modification. Shown here are XNAs discussed in this review, including both those of medical and historical relevance as well as several newly described chemistries. 2′F, 2′-fluoro; 2′OMe, 2′-O-methyl; LNA, locked nucleic acid; FANA, 2′-fluoro arabinose nucleic acid; HNA, hexitol nucleic acid; 2′MOE, 2′-O-methoxyethyl; ribuloNA, (1′-3′)-β-l-ribulo nucleic acid; TNA, α-l-threose nucleic acid; tPhoNA, 3′-2′ phosphonomethyl-threosyl nucleic acid; dXNA, 2′-deoxyxylonucleic acid; PS, phosphorothioate; phNA, alkyl phosphonate nucleic acid; PNA, peptide nucleic acid

To address these limitations, DNA and RNA analogues have been developed and evaluated for their ability to serve as useful biomaterials or functional molecules, encode genetic information, and support evolution. These synthetic genetic polymers, broadly termed xeno nucleic acids (XNAs), exhibit modified backbones, sugars, or nucleobases, and even novel bases or base pairs [10, 11]. While natural nucleic acids may also be modified to modulate their function in vivo (e.g., post-synthetic epigenetic modifications) [12–17], we constrain ourselves here to a discussion of nucleic acid congeners not found in nature. We do, however, consider certain modifications that occur sporadically in natural oligonucleotides, such as 2′OMe or C5 pyrimidine modifications, to be XNAs in the case of fully (or heavily) substituted oligonucleotides, the likes of which are not found in biology. Several new and prominent XNA chemistries are shown in Fig. 1. One attractive feature of XNAs is their generally improved chemical and biological stability [18–20]. Decoration with diverse chemical substituents (e.g., hydrophobic groups) can also yield improved properties and functionalities such as new structural motifs and enhanced target binding [21–24]. While a wide range of XNAs have been synthesized at the chemical level, representing a rich palette to draw from, their functional potential and usage is only just beginning to be probed. Here, we explore recent discoveries in the enzymatic synthesis and functional exploration of XNAs with a view towards therapeutic applications.

## Expanding the chemistry of nucleic acids

Advances in nucleic acid chemistry have enabled the development of XNAs with improved base-pairing stability over natural nucleic acids as well as enhanced activity in the context of living tissues, leading to several FDA approved nucleic acid therapeutics [25]. Research continues apace to identify novel chemistries with increased potency, bioavailability, stability, decreased toxicity, and minimal off-target effects.

An interesting development in this context is the discovery that mixed backbone chemistries can display novel emergent properties. For example, Krishnamurthy and colleagues probed the ability of XNA-RNA chimeric oligonucleotides to hybridize with their natural nucleic acid counterparts and found that the otherwise non-pairing ribuloNA can be interspersed with RNA or DNA building blocks to yield oligonucleotides with comparable or higher duplex thermal stability than natural systems and tuneable base-pairing properties [26]. The authors suggest that such a system could be used to mimic natural base-pairing rules with a reduced number of bases, storing information in the sugar in place of the GC base pair.

Similarly, the recent discovery that smaller “skinny” (pyrimidine-like) and larger “fat” (purine-like) base pairs can form stable helices challenges conventional assumptions of nucleic acid structure [27]. While the novel base pairs did not lend stability when interspersed in a natural oligonucleotide duplex, fully skinny or fat duplexes showed increased thermal stability over the corresponding natural duplexes.

In another example of XNAs reshaping classical assumptions [28], enzymatic synthesis of phosphonate nucleic acid (phNA), in which the negatively charged non-bridging oxygen of the phosphodiester linkage is replaced by an uncharged alkyl group, has enabled the demonstration of genetic function in an uncharged backbone chemistry for the first time as well as in vitro evolution of phNA aptamers [29].

As certain individual modifications become easier to synthesize and their unique properties better understood, combining two or more of these known modifications to sugars, backbones, and bases will further push the boundaries of chemical diversity in XNAs [30–35].

The development of ever more diverse chemistries raises the question: how much of what we know about natural nucleic acids translates to XNA? To what extent can we borrow design strategies from natural nucleic acids in order to rapidly develop useful new XNA chemistries? Some design rules, such as the ability of certain sequence motifs to form G-quadruplexes, have been shown to translate to TNA [36] and FANA [37]. However, the generality of such parallels remains to be explored.

## Templated synthesis of non-natural nucleic acids

The phosphoramidite approach to solid-phase DNA synthesis [38, 39] developed in the early 1980s led to the mainstream use of synthetic DNA in a myriad of applications. Similarly, improvements in the synthesis of XNAs can be expected to increase their usage and unlock new applications in the coming decades. XNA synthesis can be broadly divided into enzymatic and non-enzymatic approaches. Some widely used XNAs (e.g., 2′OMe, LNA, PS, 2′MOE) can be chemically synthesized, although yields of even the most synthetically accessible XNAs are limited to around 150 bp [40]. For other XNA chemistries, no reliable solid-phase synthetic route is known [41]. Furthermore, solid-phase synthesis depends on explicit knowledge of the sequence being synthesized. In contrast, templated synthesis enables general information transfer and is essential for the evolution of functional oligonucleotides.

### Non-enzymatic templated synthesis

Non-enzymatic templated synthesis traditionally uses activated nucleotides or short oligonucleotide building blocks that self-organize on a template via base-paring interactions and react to polymerize. Such template copying, pioneered in the 1970s [42–44], has been extended to several XNA chemistries [45–53], often in the context of prebiotic nucleic acid replication. For example, systems for templated replication of PNA pentamers [54, 55] using reductive amination are efficient enough to permit model selection experiments [56]. Similar strategies have also been exploited to assemble nucleic acids via copper-catalyzed click ligation of oligonucleotides [57, 58] and by phosphoramidate ligation [59], and the resulting backbones have shown some biocompatibility [60]. In an interesting extension of this work, nucleic acid templates have also been used to organize chemical entities by proximity for programmed reactions [61–63]. In particular, the Liu group has extended such templated synthesis approaches to chemically ligate diverse non-nucleic acid entities in precise sequences defined by a DNA template [64]. Such templated chemical syntheses allow increasingly divergent chemistries to be synthesized and replicated without constraining polymer diversity by the ability of its constituent monomers to serve as substrates for polymerases or other nucleic acid-modifying enzymes.

### Enzymatic ligation and modification of XNAs

Several DNA ligases have been shown to accept non-natural substrates and have been used alone or in conjunction with XNA polymerases to synthesize XNA oligonucleotides. Much like the aforementioned chemical ligation strategies, enzymatic ligation of pre-organized oligomers on a template allows for positioning of modified nucleotides, including multiple different modifications, at defined loci. It has recently been shown that several commercially available ligases can catalyze ligation of XNA substrates including 2′OMe, HNA, LNA, TNA, and FANA [41, 65]. Moreover, rational design and molecular modeling approaches have now resulted in the first XNA-templated XNA ligase [66]. The Liu and Hili groups have also worked extensively with ligase-mediated synthesis of modified DNA from diversely functionalized DNA 3- or 5-mers [67, 68], allowing a great variety of modifications, including hydrophobic, aliphatic, aromatic, acid, and basic moieties, to be incorporated using these short “quasicodons.” In agreement with results obtained with SOMAmer (Slow Off-rate Modified Aptamer) selections [22], they find that functionalization with nonpolar moieties appears to promote faster selection convergence and stronger aptamer binding [21]. Incorporating chemical diversity beyond that found in proteins (e.g., halogenated residues), the group isolated high affinity aptamers against PCSK9 and interleukin-6 [69]. The above strategies encapsulate the paradigm of leveraging the unique properties of nucleic acids (i.e., encoded synthesis, evolvability) coupled with an expanded set of chemical substituents to create functional molecules with therapeutic potential.

### Polymerase synthesis of XNAs

Enzymatic polymerization of nucleic acids is orders of magnitude faster and more accurate than chemical copying or synthesis and is now being pursued for next-generation DNA oligonucleotide synthesis [70, 71], with a view to supersede phosphoramidite technology. Similarly, enzymatic XNA synthesis may facilitate production of XNA oligonucleotides. XNAs with chemically conservative modifications are accepted as substrates by natural polymerases, while engineering of polymerases to accept a broader range of XNA substrates has also met with success [72, 73]. Indeed, some polymerases have proven highly adaptable to new substrates. Thermophilic B-family polymerases, especially those from *Pyrococcus* and *Thermococcus* genera, have proven especially amenable to engineering for XNA substrates [33, 74–81]. This functional plasticity may be aided by high thermostability, which promotes greater tolerance for mutations that would otherwise be excessively destabilizing. However, A-family [82–90] and mesophilic polymerases such as Phi29 [91, 92] as well as RNA polymerases [93–97] have also been engineered to accept XNA substrates with success (Table 1). However, the efficiency, fidelity, and kinetics of engineered polymerases on XNA substrates are usually compromised relative to the native enzymes and natural substrates. Often, “forcing” conditions (e.g., superstoichiometric polymerase concentrations or the presence of Mn^2+^, which in turn reduces fidelity) are needed to boost synthetic yields [106].

**Table 1:** Polymerase-mediated synthesis of XNAs

Pol Family		Polymerase	Novel Activity	Ref.
Pol A		Taq Tth Pol θ	2’F RNA 2’OME RNA 2’-azido RNA	[83, 84, 87, 88, 90, 98]
Pol B		Tgo KOD 9^°^N Pfu phi29	CeNA LNA phNA HNA FANA CyDNA 2’F RNA ANA TNA 2’azido RNA tPhoNA	[29, 33, 75, 78, 81, 82, 92]
Pol Y		(D-aa) Dpo4	L-DNA	[99–101]
Pol X		(D-aa) ASFV pol	L-DNA L-RNA	[102]
RNAP		T7 RNAP Syn5	2’F RNA 2’OMe RNA Ds-Pa UBP	[95, 97, 103, 104]
RT		HIV-RT	pyDAD-puADA UBP	[105]

Natural and engineered polymerases across of wide range of families have been used to synthesize unnatural nucleic acids. Polymerases are shown as space-filling models with primer shown in blue and templates shown in red. PDB codes are 4BWJ (Taq), 5OMF (KOD), 4G3I (Dpo4), 5HRF (ASFV), 1H38 (T7 RNAP), and 6HAK (HIV RT)

### Capitalizing on the promiscuity of natural polymerases

It is perhaps remarkable that natural polymerases can incorporate modified nucleotides at all, given their need for stringent substrate specificity and accuracy. However, at some positions in the nucleotide, modifications are readily tolerated. For example, modifications at the C5 position of pyrimidines and C7 position of N7-deaza-purines project into the duplex’s major groove and do not interfere with Watson-Crick base pairing. Therefore, even bulky adducts at these positions are generally well tolerated by polymerases [22, 107–112]. Enzymatic synthesis of nucleotides with reactive groups (e.g., for copper-catalyzed click chemistry) at these positions can allow elaborate post-synthetic modifications [113–117].

The plasticity of polymerases has also allowed researchers to carry out wholesale replacement of natural bases with close chemical analogues [118, 119]. In some cases, synthesis is efficient enough to produce modified PCR products of up to 1.5 kb [119]. Moreover, entirely new unnatural base pairs (UBPs) have been developed based on novel hydrogen bonding patterns [105, 120], hydrophobicity [121, 122], and shape complementarity [123–125]. These UBPs can be replicated by engineered and natural polymerases, demonstrating successful expansion of the genetic code, and recently achieving information encoding in an unprecedented eight-letter genetic alphabet [120].

### XNA synthesis in vivo

The synthesis and replication of XNAs in vivo represents an important frontier in XNA research towards aims such as stable encoding of unnatural amino acids and genetic orthogonality for “firewalling” synthetic biology. To this end, complete replacement of cytidine and/or thymidine with 5-substituted pyrimidine analogues in bacterial genomes has now been achieved by careful metabolic engineering and evolution [126–129].

Remarkably, Romesberg and colleagues have been able to engineer *E. coli* strains with the capacity to replicate and maintain their unnatural NaM–TPT3 base pair in both plasmid [130] and genomic [131] contexts. To achieve this, they engineered a nucleoside triphosphate transporter [132], invoked Cas9 to degrade sequences having lost the UBP, and made several tweaks to the *E. coli* DNA synthesis and repair machinery. They further demonstrated the compatibility of the UBP with translation machinery to site-specifically encode unnatural amino acids [133, 134]. Further semi-synthetic organisms with substituted genomes or the ability to propagate UBPs will likely follow these early successes [135, 136].

### Directed evolution for XNA polymerase engineering

While base modifications and even completely new base pairs are tolerated by natural polymerases to varying extents, modifications to the sugar and backbone generally prove more challenging, especially at full substitution. To improve activity with such unnatural substrates, polymerase engineering approaches, ranging from rational engineering driven by structural insights or computational analysis to screening and directed evolution, are often employed. Among these, emulsion-based methods and phage display have been especially useful [137, 138].

A range of directed evolution strategies have been employed in the field. Compartmentalized self-replication (CSR) [139–141] challenges polymerases to PCR amplify their own gene inside an emulsion compartment. The exponential enrichment of highly active clones, as opposed to simple partitioning of active variants, makes CSR a powerful method. However, it places stringent demands on polymerase performance, which are somewhat relieved in short-patch CSR (spCSR) [85] where amplification is confined to only a short section of the polymerase gene. Nested CSR allows selection of sequences or features not present in the polymerase gene itself (e.g., novel base pairs, reverse transcription of XNA templates) by using out-nesting primers with these features during the CSR amplification step [79, 86]. A further extension called compartmentalized partnered replication (CPR) can be used to select for enzymatic activities other than nucleic acid polymerization by constraining the selectable PCR reaction by the fitness of a partner gene [86, 142]. Recently, CSR has also been adapted for isothermal amplification reactions (iCSR), both at high temperatures [143] and at lower temperatures for evolution of non-thermostable enzymes [91]. Compartmentalized self-tagging (CST) [144] allows selection for much more difficult substrates or conditions; selection is based on a primer extension templated by the encoding plasmid. The biotinylated primer along with any plasmids that have been “captured” by sufficient primer extension are partitioned on streptavidin beads. CST has yielded polymerases for a range of XNAs [78], most recently for dxNA [145]. In contrast to bulk emulsification methods, microfluidic devices have been used to generate highly homogeneous emulsion droplets, enhancing the ability to accurately distinguish small incremental improvements important in stepwise selections [146, 147]. Another directed evolution strategy is phage display, whereby polymerases displayed on phage particles are challenged to extend a primer with separable (e.g., biotinylated) nucleotides [80, 89, 148]. In a notable recent use of phage display for polymerase evolution, Chen et al. identified several Taq variants capable of synthesizing and reverse transcribing 2′OMe RNA [88], an XNA of particular interest for therapeutics due to its high biostability and nuclease resistance.

### Rational engineering of XNA polymerases: structural insights

Polymerases make many key contacts with the incoming nucleotide triphosphate and primer strand during the catalytic cycle [149]. Structures of polymerases in complex with modified substrates may therefore aid in both rational engineering and retrospective understanding of XNA polymerases. Recently, Kropp et al. solved ternary structures of KlenTaq polymerase with a C5-modified cytidine at each of six successive positions [150] in the catalysis cycle, recapitulating the movement of the modified substrate through the polymerase’s active site and revealing a surprising level of flexibility in both the modified nucleotide and the interacting polymerase residues to accommodate the modification.

Further structural insight into XNA polymerization has been reported by Singh et al. [151] with structures of an engineered KlenTaq in a binary pre-incorporation and a ternary post-incorporation complex with the unnatural base pair dZ:dP. Although none of the mutated amino acids are in direct contact with primer, template, or incoming dZTP, the structure suggests that increased flexibility, particularly in the thumb subdomain, and an increased angle of closure in the finger subdomain facilitate primer elongation with the unnatural base pair.

Chim et al. recently reported ternary structures of a hyperthermophilic B-family polymerase, an engineered KOD mutant, polymerizing TNA [152]. Given the prevalent use of B-family hyperthermophiles in polymerase engineering, the structures of the apo, binary, and ternary polymerase complexes will prove useful in developing hypotheses for further work in the field. Subsequently published ternary structures of KOD and 9°N with DNA primer and template and an incoming dNTP [149, 153] suggest possible explanations for the predisposition of hyperthermophilic archaeal B-family polymerases to engineering: an unusually wide and positively charged channel between the finger and thumb subdomains may allow space for substrate modifications while maintaining strong binding to the template and high processivity. Additionally, whereas the primer template duplex adopts an A-form in the active site of A-family polymerases, it adopts a B-form in KOD, possibly contributing to accommodation of nucleotide modifications in the B-form duplex’s wider major groove.

### Rational engineering of XNA polymerases: translation of mutations across substrates and polymerases

There are numerous examples of transferable mutations across XNA substrates and polymerases. A recent report [154] details engineering of a previously described Taq mutant to better incorporate 2′ modified XNAs. From kinetic data on incorporation of different 2′ modified nucleotides, relevant mutations from the literature were chosen based on the hypothesis that the rate-limiting step may be recognition of the modified primer strand. Several mutants showed enhanced synthesis of 2′F and 2′OMe and/or reverse transcription of 2′OMe RNA. In another instance, diversification at eight “specificity determining residues” selected by computational analysis, evolutionary conservation, and literature precedent yielded improved polymerases for RNA and TNA [75]. Furthermore, testing the top mutation sets in the context of different homologous B-family polymerases evidenced their general utility as well as revealing the structural context in which they functioned best. Similarly, polymerase synthesis of increasingly diverse XNAs was achieved with a TNA polymerase by combining the TNA sugar with base modifications known to be polymerase-compatible [31, 32]. While it has been generally believed that mutation of conserved residues is highly deleterious, it is becoming increasingly apparent through this and other work that such mutations can be key in allowing activity with unnatural substrates [75, 155].

Liu et al. recently reported engineering of a polymerase to synthesize a newly described XNA, tPhoNA, with both sugar and backbone modifications [33]. Impressively, the polymerase engineering strategy consisted solely of successive introduction and evaluation of mutations at positions known to affect synthesis for other XNA substrates. Their success is a testament to the accumulating knowledge base in the field of polymerase engineering and the extraordinary ability of a small number of specific key mutations to confer an expanded substrate spectrum.

RNA polymerases have also been engineered to synthesize XNAs. It has been shown that T7 RNAP can transcribe content-expanded RNA from DNA containing the Ds-Pa UPB as well as further modified Pa bases [103]. Through a small screen of previously identified mutations, Kimoto et al. identified a T7 RNAP mutant that incorporates 2′-F uracil and cytidine triphosphates and shows improved incorporation of Pa nucleotides and their analogues in difficult sequence contexts [93]. Surprisingly, several bulky modifications of the Pa nucleotide designed to expand chemical diversity for aptamer and ribozyme selections appear to be even better substrates than the original Pa triphosphates. Similarly, a Tgo polymerase mutant previously evolved to synthesize HNA was recently shown to incorporate HNA nucleotides with various aromatic modifications on the uridine base [35]. Here too, some of the bulkier base modifications demonstrated superior incorporation.

### Reverse transcription of XNAs

Identification of enzymes capable of reverse transcribing XNAs demonstrates the potential of these divergent chemistries as genetic materials and is crucial for in vitro selections [156]. Some XNAs can be reverse transcribed by natural polymerases (e.g., TNA and FANA by Bst polymerase [157–159]). Alternately, engineering approaches must be taken [79, 88]. A small number of mutations in *Tgo* polymerase yielded a reverse transcriptase with a generally expanded substrate spectrum that can reverse transcribe several XNAs to DNA [29, 78].

### Analysis of XNAs and XNA polymerases

One difficulty in working with XNAs is that they are often incompatible with traditional methods of nucleic acid manipulation or analysis such as restriction enzymes and sequencing technologies. Thus, an important parallel effort to the development of XNAs and XNA polymerases is the development of tools to analyze them. For example, fidelity has not been evaluated in detail for many of the described XNA polymerases. A recent method for assaying fidelity reported finding that even relatively small and natural RNA modifications can significantly increase polymerase error rates [160]. Deep sequencing has also been used to investigate error profiles and polymerase read-through of templates with backbone modifications [161] and found that some commonly used isosteric modifications, such as phosphorothioates, caused significant copying errors. This fidelity data foreshadows an important limitation in enzymatic XNA synthesis that will likely need to be addressed as the field moves forward. On the other hand, the promiscuous behavior of polymerases has been leveraged to read and record the presence of epigenetic DNA and RNA modifications via misincorporation “signatures” at the modifications [162–170]. In addition, the first instance of direct sequencing of an XNA was recently reported; FANA was sequenced using nanopore technology, albeit with relatively short read lengths [171].

An XNA particularly limited by a lack of tools is L-DNA. Such “mirror image” DNA provides both complete orthogonality in macromolecule-scale features and interactions, while maintaining identical properties to DNA at the chemical level. Thus, enzymatic synthesis of L-DNA requires mirror image polymerases made of D-amino acids. Zhu and colleagues first made the D-form of the smallest known polymerase, African Swine Fever Virus polymerase X, and used it to synthesize L-DNA, showing that the polymerase was strictly enantio-specific with no crossover inhibition from D-nucleotide triphosphates [102]. The group subsequently reported synthesis of a mutant of the larger thermostable Dpo4 polymerase with D-amino acids, which enabled PCR amplification of an L-DNA product [99, 100]. Klussmann and colleagues had also reported synthesis of a D-Dpo4 mutant [101], which was used to assemble gene-length L-DNA sequences. A mirror image ligase has also been reported [172]. Mirror image nucleic acids are of immense interest for therapeutics and are being actively pursued in research and clinical development [173]. However, their usage remains limited by the arduous process of synthesizing D-polymerases and the incompatibility of L-DNA with traditional nucleic acid manipulation tools.

## Non-natural nucleic acids for therapeutic applications

Current FDA- and EMA-approved nucleic acid therapeutics (Table 2) can be broadly defined in three categories: antisense (ASO), aptamer, and most recently, siRNA. Given the inherent instability of natural nucleic acids in biological fluids, it is not surprising that every example from these categories is either fully or partly modified. Fomivirsen, a PS ASO to treat cytomegalovirus retinitis (CMV), was the first nucleic acid therapeutic to gain regulatory approval. It was subsequently withdrawn from the market due to a lack of demand as incidence of (CMV) infections decreased sharply [184]. Nevertheless, it proved an important step in demonstrating the safety and potential of nucleic acid medicines. Eteplirsen and Golodirsen are fully composed of a morpholino phosphoramidate (PMO) backbone, while Mipomersen, Nusinersen, Inotersen, and Volanesorsen feature both phosphorothioate (PS) and 2′methoxyethyl (2′MOE) modifications. Patisiran contains 2′OMe pyrimidine residues in both siRNA strands. The siRNA Givosiran also carries PS linkages and 2′F and 2′OMe modifications. Pegaptanib, while discovered as an RNA aptamer, underwent rounds of medicinal chemistry-like optimization and modification with 2′OMe and 2′F moieties to increase its in vivo stability and potency. Over 100 oligonucleotide drugs are currently in clinical trials [185], evidencing the belief and momentum behind these new therapeutic modalities able to address previously undruggable target classes.

**Table 2:** FDA-approved nucleic acid therapeutics as of February 2020

Drug name (trade name)	Target	Modifications	Mechanism	Indication	Approval	Ref.
Fomivirsen (Vitravene)	mRNA of the CMV immediate-early (IE)-2 protein	PS	ASO (translation blocking)	Cytomegalovirus retinitis (CMV)	FDA (1998) and EMA (1999) approved. FDA (2001) and EMA (2002) withdrawn	[174]
Pegaptanib (Macugen)	Vascular endothelial growth factor (VEGF165)	2′F, 2′OMe, PEG conjugate	Aptamer	Neovascular (wet) age-related macular degeneration	FDA approved (2004)	[175]
Mipomersen (Kynamro)	Apolipoprotein B-100 mRNA	2′MOE, PS, 5mC	ASO (RNase H)	Homozygous familial hypercholesterolemia	FDA approved (2013)	[176]
Eteplirsen (Exondys 51)	Exon 51 in dystrophin mRNA	PMO	ASO (splicing modulation)	Duchenne muscular dystrophy	FDA approved (2016)	[177]
Nusinersen (Spinraza)	Survival of motor neuron 2 (SMN2) pre-mRNA	2′MOE, PS, 5mC	ASO (splicing modulation)	Spinal muscular atrophy	FDA (2016) and EMA (2017) approved	[178]
Patisiran (Onpattro)	Transthyretin (TTR) mRNA	2′OMe	siRNA	Hereditary transthyretin-mediated amyloidosis	FDA and EMA approved (2018)	[179]
Inotersen (Tegsedi)	Transthyretin (TTR) mRNA	2′MOE, PS, 5mC	ASO (RNase H)	Hereditary transthyretin-mediated amyloidosis	FDA and EMA approved (2018)	[180]
Volanesorsen (Waylivra)	Apolipoprotein C_3_ (apo-CIII) mRNA	2′MOE, PS, 5mC	ASO (RNase H)	Familial chylomicronemia syndrome	EMA approved (2019)	[181]
Givosiran (Givlaari)	Aminolevulinate synthase 1 (ALAS1) mRNA	PS, 2′F, 2′OMe, GalNAc conjugate	siRNA	Acute hepatic porphyria	FDA approved (2019)	[182]
Golodirsen (Vyondys 53)	Exon 53 in dystrophin mRNA	PMO	ASO (splicing modulation)	Duchenne muscular dystrophy	FDA approved (2019)	[2, 183]

Somalogic has pioneered aptamer discovery with their SOMAmer platform [186], which employs modifications at the C5 position of pyrimidine nucleotides to append protein-like side chains to aptamer libraries, generating aptamers of high affinity and specificity for diagnostic and clinical applications.

As mentioned above, aptamers modified with 2′F and 2′OMe moieties have been selected previously, but Liu and colleagues have recently made possible direct selections in the latter backbone at full substitution using two Taq polymerase variants [104]. Similarly, direct selections of TNA have recently yielded aptamers against proteins [187] and small molecule targets [188]. Pursuant to the hypothesis that UBPs increase the sequence space and thus functional diversity of nucleic acid sequences, aptamers containing UBPs have been selected against both protein targets [189–191] and whole cells [192, 193].

In many cases, aptamers are selected as DNA or RNA sequences and then modified post-selection. Commonly, DNA and RNA aptamers are converted to their 2′ modified counterparts, producing useful affinity reagents for applications that would be incompatible with natural nucleic acids (e.g., for stability in alkaline conditions) [194]. In many cases, such post-SELEX modifications decrease affinity of the original aptamer, arguing for direct selection in XNA systems where possible. However, this is not always the case. A single base modification introduced in a thrombin aptamer increased its binding affinity by up to 7-fold [195]. In another recent report, Sullenger and colleagues modified RNA aptamers against prostate cancer cells with 2′F pyrimidines. Upon conjugation with small molecule drugs or therapeutic peptides, these aptamers displayed significant specificity and toxicity against cancerous but not normal cells [196, 197]. We direct the reader to a recent review for a more detailed panorama of modified aptamer chemistry and related technologies [198].

XNAs are also being explored for other new therapeutic modalities beyond siRNA, ASOs, and aptamers. Modified oligonucleotides capable of cleaving or ligating other oligonucleotides have been developed as fully modified “XNAzymes” [156]. In other cases, incorporation of modified nucleotides at specific positions can expand chemical functionality or otherwise enhance catalysis [199, 200]. Therapeutic XNAs are also being explored in the context of mRNAs [201], microRNAs [202], anti-gene oligonucleotides [203], and CRISPR guides [204, 205].

Delivery of oligonucleotides into the cytosol of target cells remains one of the major challengers in the development of nucleic acid therapeutics [206, 207]. Conjugation or encapsulation in lipids has demonstrated promise in improving pharmacokinetics and biodistribution [208], and cell-penetrating peptides can increase transport of biomolecules across cellular membranes [209, 210]. Other conjugations can enable targeting to specific tissues with high efficiency; *N*-acetylgalactosamine (GalNAc) has proven highly efficient for targeting biomolecules to the liver [211]. However, on the whole, effective delivery is a major challenge that remains to be addressed by the field.

## The future of unnatural nucleic acid therapeutics

As tools such as XNA polymerases become available for an increasing number and diversity of nucleic acid chemistries, it is becoming possible to explore in greater detail the molecular properties and therapeutic potential of these unnatural polymers. Several chemistries show promise in addressing the shortcomings that limit the utility of natural nucleic acids in therapeutic contexts. We anticipate that exploration in this field will lead to a steep increase in clinical-stage nucleic acid medicines in the coming decades.

## Data Availability

Not applicable.

## References

[1] Khvorova A, Watts JK (2017). The chemical evolution of oligonucleotide therapies of clinical utility. Nat Biotechnol.

[2] Shen X, Corey DR (2018). Chemistry, mechanism and clinical status of antisense oligonucleotides and duplex RNAs. Nucleic Acids Res.

[3] Burnett JC, Rossi JJ (2012). RNA-based therapeutics: current progress and future prospects. Chem Biol.

[4] Maier KE, Levy M (2016). From selection hits to clinical leads: progress in aptamer discovery. Mol Ther Methods Clin Dev.

[5] Zhou J, Rossi J (2017). Aptamers as targeted therapeutics: current potential and challenges. Nat Rev Drug Discov.

[6] Nimjee SM, White RR, Becker RC, Sullenger BA (2017). Aptamers as therapeutics. Annu Rev Pharmacol Toxicol.

[7] Rupaimoole R, Slack FJ (2017). MicroRNA therapeutics: towards a new era for the management of cancer and other diseases. Nat Rev Drug Discov.

[8] Sullenger BA, Nair S (2016). From the RNA world to the clinic. Science..

[9] Hussain W, Mahmood T, Hussain J, Ali N, Shah T, Qayyum S (2019). CRISPR/Cas system: a game changing genome editing technology, to treat human genetic diseases. Gene..

[10] Eremeeva E, Herdewijn P (2018). Non canonical genetic material. Curr Opin Biotechnol.

[11] Lee KH, Hamashima K, Kimoto M, Hirao I (2018). Genetic alphabet expansion biotechnology by creating unnatural base pairs. Curr Opin Biotechnol.

[12] Carell T, Kurz MQ, Muller M, Rossa M, Spada F (2018). Non-canonical bases in the genome: the regulatory information layer in DNA. Angew Chem Int Ed Engl..

[13] Kirnos MD, Khudyakov IY, Alexandrushkina NI, Vanyushin BF (1977). 2-Aminoadenine is an adenine substituting for a base in S-2L cyanophage DNA. Nature..

[14] Harcourt EM, Kietrys AM, Kool ET (2017). Chemical and structural effects of base modifications in messenger RNA. Nature..

[15] Frye M, Jaffrey SR, Pan T, Rechavi G, Suzuki T (2016). RNA modifications: what have we learned and where are we headed?. Nat Rev Genet.

[16] Cantara WA, Crain PF, Rozenski J, McCloskey JA, Harris KA, Zhang X (2011). The RNA modification database, RNAMDB: 2011 update. Nucleic Acids Res.

[17] Helm M, Alfonzo JD (2014). Posttranscriptional RNA modifications: playing metabolic games in a cell’s chemical Legoland. Chem Biol.

[18] Kratschmer C, Levy M (2017). Effect of chemical modifications on aptamer stability in serum. Nucleic Acid Ther..

[19] Matsunaga K, Kimoto M, Hanson C, Sanford M, Young HA, Hirao I (2015). Architecture of high-affinity unnatural-base DNA aptamers toward pharmaceutical applications. Sci Rep.

[20] Culbertson MC, Temburnikar KW, Sau SP, Liao JY, Bala S, Chaput JC (2016). Evaluating TNA stability under simulated physiological conditions. Bioorg Med Chem Lett.

[21] Lichtor PA, Chen Z, Elowe NH, Chen JC, Liu DR (2019). Side chain determinants of biopolymer function during selection and replication. Nat Chem Biol.

[22] Rohloff JC, Gelinas AD, Jarvis TC, Ochsner UA, Schneider DJ, Gold L (2014). Nucleic acid ligands with protein-like side chains: modified aptamers and their use as diagnostic and therapeutic agents. Mol Ther Nucleic Acids..

[23] Gelinas AD, Davies DR, Janjic N (2016). Embracing proteins: structural themes in aptamer-protein complexes. Curr Opin Struct Biol.

[24] Anosova I, Kowal EA, Dunn MR, Chaput JC, Van Horn WD, Egli M (2016). The structural diversity of artificial genetic polymers. Nucleic Acids Res.

[25] Smith CIE, Zain R (2019). Therapeutic oligonucleotides: state of the art. Annu Rev Pharmacol Toxicol.

[26] Efthymiou T, Gavette J, Stoop M, De Riccardis F, Froeyen M, Herdewijn P (2018). Chimeric XNA: an unconventional design for orthogonal informational systems. Chemistry..

[27] Hoshika S, Singh I, Switzer C, Molt RW, Leal NA, Kim MJ (2018). “Skinny” and “Fat” DNA: two new double helices. J Am Chem Soc.

[28] Benner SA (2017). Detecting Darwinism from molecules in the Enceladus plumes, Jupiter’s moons, and other planetary water lagoons. Astrobiology..

[29] Arangundy-Franklin S, Taylor AI, Porebski BT, Genna V, Peak-Chew S, Vaisman A (2019). A synthetic genetic polymer with an uncharged backbone chemistry based on alkyl phosphonate nucleic acids. Nat Chem.

[30] Levi-Acobas F, Katolik A, Rothlisberger P, Cokelaer T, Sarac I, Damha MJ (2019). Compatibility of 5-ethynyl-2′F-ANA UTP with in vitro selection for the generation of base-modified, nuclease resistant aptamers. Org Biomol Chem..

[31] Mei H, Chaput JC (2018). Expanding the chemical diversity of TNA with tUTP derivatives that are substrates for a TNA polymerase. Chem Commun (Camb)..

[32] Mei H, Shi C, Jimenez RM, Wang Y, Kardouh M, Chaput JC (2017). Synthesis and polymerase activity of a fluorescent cytidine TNA triphosphate analogue. Nucleic Acids Res.

[33] Liu C, Cozens C, Jaziri F, Rozenski J, Marechal A, Dumbre S (2018). Phosphonomethyl oligonucleotides as backbone-modified artificial genetic polymers. J Am Chem Soc.

[34] Jang MY, Song XP, Froeyen M, Marliere P, Lescrinier E, Rozenski J (2013). A synthetic substrate of DNA polymerase deviating from the bases, sugar, and leaving group of canonical deoxynucleoside triphosphates. Chem Biol.

[35] Renders M, Dumbre S, Abramov M, Kestemont D, Margamuljana L, Largy E (2019). Kinetic analysis of N-alkylaryl carboxamide hexitol nucleotides as substrates for evolved polymerases. Nucleic Acids Res.

[36] Liao JY, Anosova I, Bala S, Van Horn WD, Chaput JC. A parallel stranded G-quadruplex composed of threose nucleic acid (TNA). Biopolymers. 2017;107(3):e22999. 10.1002/bip.22999 27718227

[37] Assi HA, El-Khoury R, Gonzalez C, Damha MJ (2017). 2′-Fluoroarabinonucleic acid modification traps G-quadruplex and i-motif structures in human telomeric DNA. Nucleic Acids Res.

[38] Caruthers MH (1985). Gene synthesis machines: DNA chemistry and its uses. Science..

[39] Beaucage SL, Caruthers MH (1981). Deoxynucleoside phosphoramidites - a new class of key intermediates for deoxypolynucleotide synthesis. Tetrahedron Lett.

[40] Shivalingam A, Brown T (2016). Synthesis of chemically modified DNA. Biochem Soc Trans.

[41] Kestemont D, Renders M, Leonczak P, Abramov M, Schepers G, Pinheiro VB (2018). XNA ligation using T4 DNA ligase in crowding conditions. Chem Commun (Camb)..

[42] Orgel LE (1992). Molecular replication. Nature..

[43] Ninio J, Orgel LE (1978). Heteropolynucleotides as templates for non-enzymatic polymerizations. J Mol Evol.

[44] Shabarova ZA, Merenkova IN, Oretskaya TS, Sokolova NI, Skripkin EA, Alexeyeva EV (1991). Chemical ligation of DNA: the first non-enzymatic assembly of a biologically active gene. Nucleic Acids Res.

[45] Kozlov IA, De Bouvere B, Van Aerschot A, Herdewijn P, Orgel LE (1999). Efficient transfer of information from hexitol nucleic acids to RNA during nonenzymatic oligomerization. J Am Chem Soc.

[46] Sosson M, Richert C (2018). Enzyme-free genetic copying of DNA and RNA sequences. Beilstein J Org Chem.

[47] Schrum JP, Ricardo A, Krishnamurthy M, Blain JC, Szostak JW (2009). Efficient and rapid template-directed nucleic acid copying using 2′-amino-2′,3′-dideoxyribonucleoside-5′-phosphorimidazolide monomers. J Am Chem Soc.

[48] Chaput JC, Sinha S, Switzer C. 5-Propynyluracil diaminopurine: an efficient base-pair for non-enzymatic transcription of DNA. Chem Commun (Camb). 2002;2(15):1568–9. 10.1039/b204535d 12170785

[49] Hartel C, Gobel MW (2000). Substitution of adenine by purine-2,6-diamine improves the nonenzymatic oligomerization of ribonucleotides on templates containing thymidine. Helvetica chimica acta..

[50] Kozlov IA, Orgel LE (1999). Nonenzymatic oligomerization reactions on templates containing inosinic acid or diaminopurine nucleotide residues. Helvetica chimica acta.

[51] Kervio E, Sosson M, Richert C (2016). The effect of leaving groups on binding and reactivity in enzyme-free copying of DNA and RNA. Nucleic Acids Res.

[52] Izgu EC, Oh SS, Szostak JW (2016). Synthesis of activated 3′-amino-3′-deoxy-2-thio-thymidine, a superior substrate for the nonenzymatic copying of nucleic acid templates. Chem Commun (Camb)..

[53] Walton T, Pazienza L, Szostak JW (2019). Template-directed catalysis of a multistep reaction pathway for nonenzymatic RNA primer extension. Biochemistry..

[54] Kleiner RE, Brudno Y, Birnbaum ME, Liu DR (2008). DNA-templated polymerization of side-chain-functionalized peptide nucleic acid aldehydes. J Am Chem Soc.

[55] Rosenbaum DM, Liu DR (2003). Efficient and sequence-specific DNA-templated polymerization of peptide nucleic acid aldehydes. J Am Chem Soc.

[56] Brudno Y, Birnbaum ME, Kleiner RE, Liu DR (2010). An in vitro translation, selection and amplification system for peptide nucleic acids. Nat Chem Biol.

[57] Birts CN, Sanzone AP, El-Sagheer AH, Blaydes JP, Brown T, Tavassoli A (2014). Transcription of click-linked DNA in human cells. Angew Chem Int Ed Engl..

[58] El-Sagheer AH, Brown T (2010). New strategy for the synthesis of chemically modified RNA constructs exemplified by hairpin and hammerhead ribozymes. Proc Natl Acad Sci U S A.

[59] El-Sagheer AH, Brown T (2017). Single tube gene synthesis by phosphoramidate chemical ligation. Chem Commun (Camb)..

[60] Kukwikila M, Gale N, El-Sagheer AH, Brown T, Tavassoli A (2017). Assembly of a biocompatible triazole-linked gene by one-pot click-DNA ligation. Nat Chem.

[61] Percivalle C, Bartolo JF, Ladame S (2013). Oligonucleotide-templated chemical reactions: pushing the boundaries of a nature-inspired process. Org Biomol Chem.

[62] Di Pisa M, Seitz O (2017). Nucleic acid templated reactions for chemical biology. ChemMedChem..

[63] O’Reilly RK, Turberfield AJ, Wilks TR (2017). The evolution of DNA-templated synthesis as a tool for materials discovery. Acc Chem Res.

[64] Niu J, Hili R, Liu DR (2013). Enzyme-free translation of DNA into sequence-defined synthetic polymers structurally unrelated to nucleic acids. Nat Chem.

[65] McCloskey CM, Liao JY, Bala S, Chaput JC (2019). Ligase-mediated threose nucleic acid synthesis on DNA templates. ACS Synth Biol.

[66] Vanmeert M, Razzokov J, Mirza MU, Weeks SD, Schepers G, Bogaerts A (2019). Rational design of an XNA ligase through docking of unbound nucleic acids to toroidal proteins. Nucleic Acids Res.

[67] Kong D, Lei Y, Yeung W, Hili R (2016). Enzymatic synthesis of sequence-defined synthetic nucleic acid polymers with diverse functional groups. Angew Chem Int Ed Engl..

[68] Hili R, Niu J, Liu DR (2013). DNA ligase-mediated translation of DNA into densely functionalized nucleic acid polymers. J Am Chem Soc.

[69] Chen Z, Lichtor PA, Berliner AP, Chen JC, Liu DR (2018). Evolution of sequence-defined highly functionalized nucleic acid polymers. Nat Chem.

[70] Perkel JM (2019). The race for enzymatic DNA synthesis heats up. Nature..

[71] Palluk S, Arlow DH, de Rond T, Barthel S, Kang JS, Bector R (2018). De novo DNA synthesis using polymerase-nucleotide conjugates. Nat Biotechnol.

[72] Eremeeva E, Herdewijn P. Enzymatic synthesis using polymerases of modified nucleic acids and genes. In: Fernández-Lucas J, editor. Enzymatic and Chemical Synthesis of Nucleic Acid Derivatives. Weinheim: Wiley-VCH; 2018. p. 159–194.

[73] Houlihan G, Arangundy-Franklin S, Holliger P (2017). Engineering and application of polymerases for synthetic genetics. Curr Opin Biotechnol.

[74] Hoshino H, Kasahara Y, Fujita H, Kuwahara M, Morihiro K, Tsunoda SI (2016). Consecutive incorporation of functionalized nucleotides with amphiphilic side chains by novel KOD polymerase mutant. Bioorg Med Chem Lett.

[75] Dunn MR, Otto C, Fenton KE, Chaput JC (2016). Improving polymerase activity with unnatural substrates by sampling mutations in homologous protein architectures. ACS Chem Biol.

[76] Horhota A, Zou K, Ichida JK, Yu B, McLaughlin LW, Szostak JW (2005). Kinetic analysis of an efficient DNA-dependent TNA polymerase. J Am Chem Soc.

[77] Cozens C, Mutschler H, Nelson GM, Houlihan G, Taylor AI, Holliger P (2015). Enzymatic synthesis of nucleic acids with defined regioisomeric 2′-5′ linkages. Angew Chem Int Ed Engl..

[78] Pinheiro VB, Taylor AI, Cozens C, Abramov M, Renders M, Zhang S (2012). Synthetic genetic polymers capable of heredity and evolution. Science..

[79] Ellefson JW, Gollihar J, Shroff R, Shivram H, Iyer VR, Ellington AD (2016). Synthetic evolutionary origin of a proofreading reverse transcriptase. Science..

[80] Xia G, Chen L, Sera T, Fa M, Schultz PG, Romesberg FE (2002). Directed evolution of novel polymerase activities: mutation of a DNA polymerase into an efficient RNA polymerase. Proc Natl Acad Sci U S A.

[81] Cozens C, Pinheiro VB, Vaisman A, Woodgate R, Holliger P (2012). A short adaptive path from DNA to RNA polymerases. Proc Natl Acad Sci U S A.

[82] Ramsay N, Jemth AS, Brown A, Crampton N, Dear P, Holliger P (2010). CyDNA: synthesis and replication of highly Cy-dye substituted DNA by an evolved polymerase. J Am Chem Soc.

[83] Ghadessy FJ, Ramsay N, Boudsocq F, Loakes D, Brown A, Iwai S (2004). Generic expansion of the substrate spectrum of a DNA polymerase by directed evolution. Nat Biotechnol.

[84] Loakes D, Gallego J, Pinheiro VB, Kool ET, Holliger P (2009). Evolving a polymerase for hydrophobic base analogues. J Am Chem Soc.

[85] Ong JL, Loakes D, Jaroslawski S, Too K, Holliger P (2006). Directed evolution of DNA polymerase, RNA polymerase and reverse transcriptase activity in a single polypeptide. J Mol Biol.

[86] Laos R, Shaw R, Leal NA, Gaucher E, Benner S (2013). Directed evolution of polymerases to accept nucleotides with nonstandard hydrogen bond patterns. Biochemistry..

[87] Schultz HJ, Gochi AM, Chia HE, Ogonowsky AL, Chiang S, Filipovic N (2015). Taq DNA polymerase mutants and 2′-modified sugar recognition. Biochemistry..

[88] Chen T, Hongdilokkul N, Liu Z, Adhikary R, Tsuen SS, Romesberg FE (2016). Evolution of thermophilic DNA polymerases for the recognition and amplification of C2′-modified DNA. Nat Chem.

[89] Delespaul W, Peeters Y, Herdewijn P, Robben J (2015). A novel helper phage for HaloTag-mediated co-display of enzyme and substrate on phage. Biochem Biophys Res Commun.

[90] Randrianjatovo-Gbalou I, Rosario S, Sismeiro O, Varet H, Legendre R, Coppee JY (2018). Enzymatic synthesis of random sequences of RNA and RNA analogues by DNA polymerase theta mutants for the generation of aptamer libraries. Nucleic Acids Res.

[91] Povilaitis T, Alzbutas G, Sukackaite R, Siurkus J, Skirgaila R (2016). In vitro evolution of phi29 DNA polymerase using isothermal compartmentalized self replication technique. Protein Eng Des Sel.

[92] Torres LL, Pinheiro VB (2018). Xenobiotic nucleic acid (XNA) synthesis by Phi29 DNA polymerase. Curr Protoc Chem Biol..

[93] Kimoto M, Meyer AJ, Hirao I, Ellington AD (2017). Genetic alphabet expansion transcription generating functional RNA molecules containing a five-letter alphabet including modified unnatural and natural base nucleotides by thermostable T7 RNA polymerase variants. Chem Commun (Camb)..

[94] Meyer AJ, Garry DJ, Hall B, Byrom MM, McDonald HG, Yang X (2015). Transcription yield of fully 2′-modified RNA can be increased by the addition of thermostabilizing mutations to T7 RNA polymerase mutants. Nucleic Acids Res.

[95] Zhu B, Hernandez A, Tan M, Wollenhaupt J, Tabor S, Richardson CC (2015). Synthesis of 2′-fluoro RNA by Syn5 RNA polymerase. Nucleic Acids Res.

[96] Chelliserrykattil J, Ellington AD (2004). Evolution of a T7 RNA polymerase variant that transcribes 2′-O-methyl RNA. Nat Biotechnol.

[97] Ibach J, Dietrich L, Koopmans KR, Nobel N, Skoupi M, Brakmann S (2013). Identification of a T7 RNA polymerase variant that permits the enzymatic synthesis of fully 2′-O-methyl-modified RNA. J Biotechnol.

[98] Kent T, Rusanov TD, Hoang TM, Velema WA, Krueger AT, Copeland WC (2016). DNA polymerase theta specializes in incorporating synthetic expanded-size (xDNA) nucleotides. Nucleic Acids Res.

[99] Jiang W, Zhang B, Fan C, Wang M, Wang J, Deng Q (2017). Mirror-image polymerase chain reaction. Cell Discov..

[100] Xu W, Jiang W, Wang J, Yu L, Chen J, Liu X (2017). Total chemical synthesis of a thermostable enzyme capable of polymerase chain reaction. Cell Discov.

[101] Pech A, Achenbach J, Jahnz M, Schulzchen S, Jarosch F, Bordusa F (2017). A thermostable d-polymerase for mirror-image PCR. Nucleic Acids Res.

[102] Wang Z, Xu W, Liu L, Zhu TF (2016). A synthetic molecular system capable of mirror-image genetic replication and transcription. Nat Chem.

[103] Hirao I, Kimoto M, Mitsui T, Fujiwara T, Kawai R, Sato A (2006). An unnatural hydrophobic base pair system: site-specific incorporation of nucleotide analogs into DNA and RNA. Nat Methods.

[104] Liu Z, Chen T, Romesberg FE (2017). Evolved polymerases facilitate selection of fully 2′-OMe-modified aptamers. Chem Sci.

[105] Sismour AM, Lutz S, Park JH, Lutz MJ, Boyer PL, Hughes SH (2004). PCR amplification of DNA containing non-standard base pairs by variants of reverse transcriptase from human immunodeficiency virus-1. Nucleic Acids Res.

[106] Cozens C, Pinheiro VB (2018). XNA synthesis and reverse transcription by engineered thermophilic polymerases. Curr Protoc Chem Biol.

[107] Bergen K, Steck AL, Strutt S, Baccaro A, Welte W, Diederichs K (2012). Structures of KlenTaq DNA polymerase caught while incorporating C5-modified pyrimidine and C7-modified 7-deazapurine nucleoside triphosphates. J Am Chem Soc.

[108] Obeid S, Baccaro A, Welte W, Diederichs K, Marx A (2010). Structural basis for the synthesis of nucleobase modified DNA by Thermus aquaticus DNA polymerase. Proc Natl Acad Sci U S A.

[109] Cahova H, Panattoni A, Kielkowski P, Fanfrlik J, Hocek M (2016). 5-Substituted pyrimidine and 7-substituted 7-deazapurine dNTPs as substrates for DNA polymerases in competitive primer extension in the presence of natural dNTPs. ACS Chem Biol.

[110] Hottin A, Marx A (2016). Structural insights into the processing of nucleobase-modified nucleotides by DNA polymerases. Acc Chem Res.

[111] Balintova J, Welter M, Marx A (2018). Antibody-nucleotide conjugate as a substrate for DNA polymerases. Chem Sci.

[112] Welter M, Verga D, Marx A (2016). Sequence-specific incorporation of enzyme-nucleotide chimera by DNA polymerases. Angew Chem Int Ed Engl..

[113] Kath-Schorr S (2016). Cycloadditions for studying nucleic acids. Top Curr Chem (Cham).

[114] Pfeiffer F, Tolle F, Rosenthal M, Brandle GM, Ewers J, Mayer G (2018). Identification and characterization of nucleobase-modified aptamers by click-SELEX. Nat Protoc.

[115] Tolle F, Brandle GM, Matzner D, Mayer G (2015). A versatile approach towards nucleobase-modified aptamers. Angew Chem Int Ed Engl..

[116] Temme JS, MacPherson IS, DeCourcey JF, Krauss IJ (2014). High temperature SELMA: evolution of DNA-supported oligomannose clusters which are tightly recognized by HIV bnAb 2G12. J Am Chem Soc.

[117] Eggert F, Kath-Schorr S (2016). A cyclopropene-modified nucleotide for site-specific RNA labeling using genetic alphabet expansion transcription. Chem Commun (Camb).

[118] Eremeeva E, Abramov M, Marliere P, Herdewijn P (2016). The 5-chlorouracil:7-deazaadenine base pair as an alternative to the dT:dA base pair. Org Biomol Chem..

[119] Eremeeva E, Abramov M, Margamuljana L, Herdewijn P (2017). Base-modified nucleic acids as a powerful tool for synthetic biology and biotechnology. Chemistry..

[120] Hoshika S, Leal NA, Kim MJ, Kim MS, Karalkar NB, Kim HJ (2019). Hachimoji DNA and RNA: a genetic system with eight building blocks. Science..

[121] Li L, Degardin M, Lavergne T, Malyshev DA, Dhami K, Ordoukhanian P (2014). Natural-like replication of an unnatural base pair for the expansion of the genetic alphabet and biotechnology applications. J Am Chem Soc.

[122] Leconte AM, Hwang GT, Matsuda S, Capek P, Hari Y, Romesberg FE (2008). Discovery, characterization, and optimization of an unnatural base pair for expansion of the genetic alphabet. J Am Chem Soc.

[123] Kimoto M, Kawai R, Mitsui T, Yokoyama S, Hirao I (2009). An unnatural base pair system for efficient PCR amplification and functionalization of DNA molecules. Nucleic Acids Res.

[124] Yamashige R, Kimoto M, Takezawa Y, Sato A, Mitsui T, Yokoyama S (2012). Highly specific unnatural base pair systems as a third base pair for PCR amplification. Nucleic Acids Res.

[125] Okamoto I, Miyatake Y, Kimoto M, Hirao I (2016). High fidelity, efficiency and functionalization of Ds-Px unnatural base pairs in PCR amplification for a genetic alphabet expansion system. ACS Synth Biol.

[126] Marliere P, Patrouix J, Doring V, Herdewijn P, Tricot S, Cruveiller S (2011). Chemical evolution of a bacterium’s genome. Angew Chem Int Ed Engl..

[127] Mehta AP, Li H, Reed SA, Supekova L, Javahishvili T, Schultz PG (2016). Replacement of thymidine by a modified base in the Escherichia coli genome. J Am Chem Soc.

[128] Mehta AP, Li H, Reed SA, Supekova L, Javahishvili T, Schultz PG (2016). Replacement of 2′-deoxycytidine by 2′-deoxycytidine analogues in the E. coli genome. J Am Chem Soc.

[129] Eremeeva E, Abramov M, Margamuljana L, Rozenski J, Pezo V, Marliere P (2016). Chemical morphing of DNA containing four noncanonical bases. Angew Chem Int Ed Engl..

[130] Zhang Y, Lamb BM, Feldman AW, Zhou AX, Lavergne T, Li L (2017). A semisynthetic organism engineered for the stable expansion of the genetic alphabet. Proc Natl Acad Sci U S A.

[131] Ledbetter MP, Karadeema RJ, Romesberg FE (2018). Reprograming the replisome of a semisynthetic organism for the expansion of the genetic alphabet. J Am Chem Soc.

[132] Feldman AW, Fischer EC, Ledbetter MP, Liao JY, Chaput JC, Romesberg FE (2018). A tool for the import of natural and unnatural nucleoside triphosphates into bacteria. J Am Chem Soc.

[133] Zhang Y, Ptacin JL, Fischer EC, Aerni HR, Caffaro CE, San Jose K (2017). A semi-synthetic organism that stores and retrieves increased genetic information. Nature..

[134] Dien VT, Holcomb M, Feldman AW, Fischer EC, Dwyer TJ, Romesberg FE (2018). Progress toward a semi-synthetic organism with an unrestricted expanded genetic alphabet. J Am Chem Soc.

[135] Pezo V, Hassan C, Louis D, Sargueil B, Herdewijn P, Marliere P (2018). Metabolic recruitment and directed evolution of nucleoside triphosphate uptake in Escherichia coli. ACS Synth Biol.

[136] Bande O, Abu El Asrar R, Braddick D, Dumbre S, Pezo V, Schepers G, et al. Isoguanine and 5-methyl-isocytosine bases, in vitro and in vivo. Chemistry. 2015;21(13):5009–5022. 10.1002/chem.201406392 PMC4531829 25684598

[137] Houlihan G, Arangundy-Franklin S, Holliger P (2017). Exploring the chemistry of genetic information storage and propagation through polymerase engineering. Acc Chem Res.

[138] Tizei PA, Csibra E, Torres L, Pinheiro VB (2016). Selection platforms for directed evolution in synthetic biology. Biochem Soc Trans.

[139] Ghadessy FJ, Holliger P (2007). Compartmentalized self-replication: a novel method for the directed evolution of polymerases and other enzymes. Methods Mol Biol.

[140] Ghadessy FJ, Ong JL, Holliger P (2001). Directed evolution of polymerase function by compartmentalized self-replication. Proc Natl Acad Sci U S A.

[141] Abil Z, Ellington AD (2018). Compartmentalized self-replication for evolution of a DNA polymerase. Curr Protoc Chem Biol..

[142] Aye SL, Fujiwara K, Ueki A, Doi N (2018). Engineering of DNA polymerase I from Thermus thermophilus using compartmentalized self-replication. Biochem Biophys Res Commun.

[143] Milligan JN, Shroff R, Garry DJ, Ellington AD (2018). Evolution of a thermophilic strand-displacing polymerase using high-temperature isothermal compartmentalized self-replication. Biochemistry..

[144] Pinheiro VB, Arangundy-Franklin S, Holliger P (2014). Compartmentalized self-tagging for in vitro-directed evolution of XNA polymerases. Curr Protoc Nucleic Acid Chem.

[145] Bauwens B, Rozenski J, Herdewijn P, Robben J (2018). A single amino acid substitution in Therminator DNA polymerase increases incorporation efficiency of deoxyxylonucleotides. Chembiochem..

[146] Larsen AC, Dunn MR, Hatch A, Sau SP, Youngbull C, Chaput JC (2016). A general strategy for expanding polymerase function by droplet microfluidics. Nat Commun.

[147] Nikoomanzar A, Vallejo D, Chaput JC (2019). Elucidating the determinants of polymerase specificity by microfluidic-based deep mutational scanning. ACS Synth Biol.

[148] Jestin JL, Kristensen P, Winter G (1999). A method for the selection of catalytic activity using phage display and proximity coupling. Angew Chem Int Ed Engl.

[149] Kropp HM, Betz K, Wirth J, Diederichs K, Marx A (2017). Crystal structures of ternary complexes of archaeal B-family DNA polymerases. PLoS One.

[150] Kropp HM, Durr SL, Peter C, Diederichs K, Marx A (2018). Snapshots of a modified nucleotide moving through the confines of a DNA polymerase. Proc Natl Acad Sci U S A.

[151] Singh I, Laos R, Hoshika S, Benner SA, Georgiadis MM (2018). Snapshots of an evolved DNA polymerase pre- and post-incorporation of an unnatural nucleotide. Nucleic Acids Res.

[152] Chim N, Shi C, Sau SP, Nikoomanzar A, Chaput JC (2017). Structural basis for TNA synthesis by an engineered TNA polymerase. Nat Commun.

[153] Zhang Y, Kleiner RE (2019). A metabolic engineering approach to incorporate modified pyrimidine nucleosides into cellular RNA. J Am Chem Soc.

[154] Rosenblum SL, Weiden AG, Lewis EL, Ogonowsky AL, Chia HE, Barrett SE (2017). Design and discovery of new combinations of mutant DNA polymerases and modified DNA substrates. Chembiochem..

[155] Raghunathan G, Marx A (2019). Identification of Thermus aquaticus DNA polymerase variants with increased mismatch discrimination and reverse transcriptase activity from a smart enzyme mutant library. Sci Rep.

[156] Taylor AI, Pinheiro VB, Smola MJ, Morgunov AS, Peak-Chew S, Cozens C (2015). Catalysts from synthetic genetic polymers. Nature..

[157] Dunn MR, Chaput JC (2016). Reverse transcription of threose nucleic acid by a naturally occurring DNA polymerase. Chembiochem..

[158] Wang Y, Ngor AK, Nikoomanzar A, Chaput JC (2018). Evolution of a general RNA-cleaving FANA enzyme. Nat Commun.

[159] Jackson LN, Chim N, Shi C, Chaput JC (2019). Crystal structures of a natural DNA polymerase that functions as an XNA reverse transcriptase. Nucleic Acids Res.

[160] Potapov V, Fu X, Dai N, Correa IR, Tanner NA, Ong JL (2018). Base modifications affecting RNA polymerase and reverse transcriptase fidelity. Nucleic Acids Res.

[161] Shivalingam A, Tyburn AE, El-Sagheer AH, Brown T (2017). Molecular requirements of high-fidelity replication-competent DNA backbones for orthogonal chemical ligation. J Am Chem Soc.

[162] Aschenbrenner J, Werner S, Marchand V, Adam M, Motorin Y, Helm M (2018). Engineering of a DNA polymerase for direct m (6) A sequencing. Angew Chem Int Ed Engl..

[163] Blatter N, Bergen K, Nolte O, Welte W, Diederichs K, Mayer J (2013). Structure and function of an RNA-reading thermostable DNA polymerase. Angew Chem Int Ed Engl..

[164] Aschenbrenner J, Marx A (2016). Direct and site-specific quantification of RNA 2′-O-methylation by PCR with an engineered DNA polymerase. Nucleic Acids Res.

[165] Hong T, Yuan Y, Chen Z, Xi K, Wang T, Xie Y (2018). Precise antibody-independent m6A identification via 4SedTTP-involved and FTO-assisted strategy at single-nucleotide resolution. J Am Chem Soc.

[166] Wyss LA, Nilforoushan A, Williams DM, Marx A, Sturla SJ (2016). The use of an artificial nucleotide for polymerase-based recognition of carcinogenic O6-alkylguanine DNA adducts. Nucleic Acids Res.

[167] Betz K, Nilforoushan A, Wyss LA, Diederichs K, Sturla SJ, Marx A (2017). Structural basis for the selective incorporation of an artificial nucleotide opposite a DNA adduct by a DNA polymerase. Chem Commun (Camb)..

[168] von Watzdorf J, Marx A (2016). 6-Substituted 2-aminopurine-2′-deoxyribonucleoside 5′-triphosphates that trace cytosine methylation. Chembiochem..

[169] von Watzdorf J, Leitner K, Marx A (2016). Modified nucleotides for discrimination between cytosine and the epigenetic marker 5-methylcytosine. Angew Chem Int Ed Engl..

[170] Huber C, von Watzdorf J, Marx A (2016). 5-methylcytosine-sensitive variants of Thermococcus kodakaraensis DNA polymerase. Nucleic Acids Res.

[171] Yan S, Li X, Zhang P, Wang Y, Chen HY, Huang S (2019). Direct sequencing of 2′-deoxy-2′-fluoroarabinonucleic acid (FANA) using nanopore-induced phase-shift sequencing (NIPSS). Chem Sci.

[172] Weidmann J, Schnolzer M, Dawson PE, Hoheisel JD (2019). Copying life: synthesis of an enzymatically active mirror-image DNA-ligase made of D-amino acids. Cell Chem Biol.

[173] Vater A, Klussmann S (2015). Turning mirror-image oligonucleotides into drugs: the evolution of Spiegelmer((R)) therapeutics. Drug Discov Today.

[174] Stein CA, Castanotto D (2017). FDA-approved oligonucleotide therapies in 2017. Mol Ther.

[175] Ng EW, Shima DT, Calias P, Cunningham ET, Guyer DR, Adamis AP (2006). Pegaptanib, a targeted anti-VEGF aptamer for ocular vascular disease. Nat Rev Drug Discov.

[176] Parham JS, Goldberg AC (2019). Mipomersen and its use in familial hypercholesterolemia. Expert Opin Pharmacother.

[177] Charleston JS, Schnell FJ, Dworzak J, Donoghue C, Lewis S, Chen L (2018). Eteplirsen treatment for Duchenne muscular dystrophy: exon skipping and dystrophin production. Neurology..

[178] Goodkey K, Aslesh T, Maruyama R, Yokota T (1828). Nusinersen in the treatment of spinal muscular atrophy. Methods Mol Biol.

[179] Adams D, Gonzalez-Duarte A, O’Riordan WD, Yang CC, Ueda M, Kristen AV (2018). Patisiran, an RNAi therapeutic, for hereditary transthyretin amyloidosis. N Engl J Med.

[180] Benson MD, Waddington-Cruz M, Berk JL, Polydefkis M, Dyck PJ, Wang AK (2018). Inotersen treatment for patients with hereditary transthyretin amyloidosis. N Engl J Med.

[181] Paik J, Duggan S (2019). Volanesorsen: first global approval. Drugs..

[182] Scott LJ (2020). Givosiran: first approval. Drugs..

[183] Aartsma-Rus A, Corey DR. The 10th oligonucleotide therapy approved: golodirsen for Duchenne muscular dystrophy. Nucleic Acid Ther. 2020. 10.1089/nat.2020.0845 PMC7133412 32043902

[184] Port AD, Alabi RO, Koenig L, Gupta MP (2018). Cytomegalovirus retinitis in the post-cART era. Curr Ophthalmol Rep.

[185] Damha MJ (2019). Exciting times in the field of nucleic acid therapeutics. Trends Mol Med.

[186] Gold L, Ayers D, Bertino J, Bock C, Bock A, Brody EN (2010). Aptamer-based multiplexed proteomic technology for biomarker discovery. PLoS One.

[187] Mei H, Liao JY, Jimenez RM, Wang Y, Bala S, McCloskey C (2018). Synthesis and evolution of a threose nucleic acid aptamer bearing 7-deaza-7-substituted guanosine residues. J Am Chem Soc.

[188] Rangel AE, Chen Z, Ayele TM, Heemstra JM (2018). In vitro selection of an XNA aptamer capable of small-molecule recognition. Nucleic Acids Res.

[189] Kimoto M, Nakamura M, Hirao I (2016). Post-ExSELEX stabilization of an unnatural-base DNA aptamer targeting VEGF165 toward pharmaceutical applications. Nucleic Acids Res.

[190] Biondi E, Lane JD, Das D, Dasgupta S, Piccirilli JA, Hoshika S (2016). Laboratory evolution of artificially expanded DNA gives redesignable aptamers that target the toxic form of anthrax protective antigen. Nucleic Acids Res.

[191] Matsunaga KI, Kimoto M, Hirao I (2017). High-affinity DNA aptamer generation targeting von Willebrand factor A1-domain by genetic alphabet expansion for systematic evolution of ligands by exponential enrichment using two types of libraries composed of five different bases. J Am Chem Soc.

[192] Zhang L, Yang Z, Le Trinh T, Teng IT, Wang S, Bradley KM (2016). Aptamers against cells overexpressing glypican 3 from expanded genetic systems combined with cell engineering and laboratory evolution. Angew Chem Int Ed Engl..

[193] Futami K, Kimoto M, Lim YWS, Hirao I (2019). Genetic alphabet expansion provides versatile specificities and activities of unnatural-base DNA aptamers targeting cancer cells. Mol Ther Nucleic Acids.

[194] Inomata E, Tashiro E, Miyakawa S, Nakamura Y, Akita K (2018). Alkaline-tolerant RNA aptamers useful to purify acid-sensitive antibodies in neutral conditions. Biochimie..

[195] Dolot R, Lam CH, Sierant M, Zhao Q, Liu FW, Nawrot B (2018). Crystal structures of thrombin in complex with chemically modified thrombin DNA aptamers reveal the origins of enhanced affinity. Nucleic Acids Res.

[196] Powell Gray B, Kelly L, Ahrens DP, Barry AP, Kratschmer C, Levy M (2018). Tunable cytotoxic aptamer-drug conjugates for the treatment of prostate cancer. Proc Natl Acad Sci U S A.

[197] Romanelli A, Affinito A, Avitabile C, Catuogno S, Ceriotti P, Iaboni M (2018). An anti-PDGFRbeta aptamer for selective delivery of small therapeutic peptide to cardiac cells. PLoS One.

[198] Rothlisberger P, Hollenstein M (2018). Aptamer chemistry. Adv Drug Deliv Rev.

[199] Wang Y, Liu E, Lam CH, Perrin DM (2018). A densely modified M (2+)-independent DNAzyme that cleaves RNA efficiently with multiple catalytic turnover. Chem Sci.

[200] Chakravarthy M, Aung-Htut MT, Le BT, Veedu RN (2017). Novel chemically-modified DNAzyme targeting integrin alpha-4 RNA transcript as a potential molecule to reduce inflammation in multiple sclerosis. Sci Rep.

[201] Sergeeva OV, Koteliansky VE, Zatsepin TS (2016). mRNA-based therapeutics - advances and perspectives. Biochemistry (Mosc).

[202] Bajan S, Hutvagner G. RNA-based therapeutics: from antisense oligonucleotides to miRNAs. Cells. 2020;9(1):137. 10.3390/cells9010137 PMC7016530 31936122

[203] Taniguchi Y, Sasaki S (2012). An efficient antigene activity and antiproliferative effect by targeting the Bcl-2 or survivin gene with triplex forming oligonucleotides containing a W-shaped nucleoside analogue (WNA-betaT). Org Biomol Chem..

[204] Rueda FO, Bista M, Newton MD, Goeppert AU, Cuomo ME, Gordon E (2017). Mapping the sugar dependency for rational generation of a DNA-RNA hybrid-guided Cas9 endonuclease. Nat Commun.

[205] O’Reilly D, Kartje ZJ, Ageely EA, Malek-Adamian E, Habibian M, Schofield A (2019). Extensive CRISPR RNA modification reveals chemical compatibility and structure-activity relationships for Cas9 biochemical activity. Nucleic Acids Res.

[206] Johannes L, Lucchino M (2018). Current challenges in delivery and cytosolic translocation of therapeutic RNAs. Nucleic Acid Ther.

[207] Juliano RL (2016). The delivery of therapeutic oligonucleotides. Nucleic Acids Res.

[208] Craig K, Abrams M, Amiji M (2018). Recent preclinical and clinical advances in oligonucleotide conjugates. Expert Opin Drug Deliv.

[209] Derakhshankhah H, Jafari S (2018). Cell penetrating peptides: a concise review with emphasis on biomedical applications. Biomed Pharmacother.

[210] McClorey G, Banerjee S. Cell-penetrating peptides to enhance delivery of oligonucleotide-based therapeutics. Biomedicines. 2018;6(2):51. 10.3390/biomedicines6020051 PMC6027240 29734750

[211] Springer AD, Dowdy SF (2018). GalNAc-siRNA conjugates: leading the way for delivery of RNAi therapeutics. Nucleic Acid Ther..

